# Letrozole and the Traditional Chinese Medicine, Shaofu Zhuyu Decoction, Reduce Endometriotic Disease Progression in Rats: A Potential Role for Gut Microbiota

**DOI:** 10.1155/2020/3687498

**Published:** 2020-07-20

**Authors:** Ying Cao, Chunhua Jiang, Yongsen Jia, Dingjie Xu, Yanyan Yu

**Affiliations:** ^1^College of Traditional Chinese Medicine, North China University of Science and Technology, Tangshan, China; ^2^Reproductive Endocrinology Center, Dongfang Hospital, Beijing University of Chinese Medicine, Beijing, China

## Abstract

We previously showed that the Chinese herbal medicine, Shaofu Zhuyu decoction (SFZYD), shrank the size of endometriotic lesions in rats with endometriosis. We therefore conducted the present study to investigate the effects of letrozole and SFZYD on gut microbiota in endometriotic rats. Rats were divided into four groups: a blank group, model group, letrozole group, and SFZY group. Ectopic lesion size and COX-2 expression in the endometrium and endometriotic lesions were compared, and the community of gut microbiota was detected using 16S rRNA gene sequencing. Both letrozole and SFZYD reduced the size of ectopic lesions as well as lowered the expression of COX-2, thus reducing the inflammatory response. Compared with the blank group, the *α*-diversity of gut microbiota in endometriotic rats decreased, the *Firmicutes/Bacteroidetes* ratio increased, and the abundance of *Ruminococcaceae* was reduced. The *α*-diversity of gut microbiota in the letrozole group was similar to that in the model group, but the *Firmicutes/Bacteroidetes* ratio was diminished. The *α*-diversity in the SFZY group was similar to that in the blank group, the *Firmicutes/Bacteroidetes* ratio was attenuated, and the abundance of *Ruminococcaceae* was elevated compared with the model group. These results indicated that the therapeutic mechanisms of both letrozole and SFZYD were related to the restoration of gut microbiota.

## 1. Introduction

Endometriosis (EMs) is an estrogen-dependent disease in which endometrial glands and stromal tissues are implanted outside the uterine cavity. The incidence of EMs is 10% in women of childbearing age and up to 30–45% in infertile women [1]. The primary manifestations include dysmenorrhea, chronic pelvic pain, and infertility that seriously affect the quality of life of patients. Although there are many etiologic theories regarding EMs, they cannot adequately explain the occurrence and development of the disease. Moreover, its arcane etiology is an important reason hindering research on treatment for EMs.

According to the ectopic implantation theory accepted by most scholars, EMs originated from the shedding of endometrial debris that then enters the pelvic cavity with countercurrent menstrual blood flow [2]. The immune cells in the pelvic cavity are presumed to eliminate these cells, but the endometrial cells survive and lead to a continuous inflammatory status in the pelvic cavity due to the abnormal immunity in this region [3, 4]. In fact, EMs is believed to be a chronic inflammatory disease from an increasing number of studies [5, 6]. Investigators have demonstrated an increase in the number of activated macrophages and proinflammatory cytokines and angiogenic factors in the pelvic fluid of EMs patients [7, 8], which could provide a favorable environment for ectopic endometrial implantation [8]. Compared with normal pelvic fluid, the pelvic fluid in EMs patients promotes the expression of the vascular endothelial growth factor (VEGF) and urokinase plasminogen activator (uPA) in endometrial cells [9]. Lipopolysaccharide (LPS), a common endotoxin, is at a much higher concentration in the menstrual blood of EMs patients relative to women without EMs, and the combination of LPS and toll-like receptor 4 (TLR4) can promote the proliferation of eutopic endometrial stromal cells. The quantity of *E. coli* (i.e., the principal source of LPS in the menstrual blood of EMs patients) is also much higher than that in the normal population [10].

The gut microbiota is known to be intimately involved in inflammatory responses. Karmarkar and Rock found that gut microbiota activated neutrophils via the myeloid differentiation primary response 88 (MyD88) pathway, which was a prerequisite for a pelvic inflammatory response [11]. Emani et al. found that abnormal gut microbiota weakened the function of the intestinal barrier, thereby leading to the leakage of bacteria into the pelvic cavity [12], and simultaneously facilitated the translocation of LPS from the intestinal epithelium to the pelvic cavity [13]. In addition, gut microbiota can affect the estrogen concentrations in circulation [14]. Therefore, researchers have increasingly focused on the relationship between the gut microbiota and the progression of EMs. Previous studies have confirmed that a mouse model of EMs exhibits dysbiosis of the gut microbiota [15]. Wide-spectrum antibiotics (e.g., vancomycin, neomycin, metronidazole, and ampicillin) inhibited the growth of ectopic lesions in EMs mice, and the oral gavage of feces from mice with EMs restored the growth of endometriotic lesions and inflammation in metronidazole-treated mice [16].

Letrozole, as a third-generation aromatase inhibitor, can inhibit the production of circulating and local estrogen and has been used in the experimental treatment of EMs in animal and clinical studies [17]. Shaofu Zhuyu decoction (SFZYD) is a classic prescription of the traditional Chinese medicine commonly used in treating dysmenorrhea; it originated from the *Correction of the Errors of Medical Wor*ks by Wang Qingren, a physician in the Qing Dynasty. SFZYD is now widely used in TCM therapy for endometriosis. Our previous study showed that SFZYD inhibited cellular proliferation, promoted apoptosis, and reduced angiogenesis in ectopic endometrial tissues, thus playing a role in the treatment of endometriosis [18]. The purpose of the present study is to investigate our hypothesis that letrozole and SFZYD act on gut microbiota to inhibit the progression of lesions in rats with EMs.

## 2. Materials and Methods

### 2.1. Chemicals and Reagents

Estradiol valerate (Delpharm Lille S.A.S., France) and letrozole (Hengrui Medicine, China) were purchased from the eye hospital of the China Academy of Chinese Medical Sciences. Pentobarbital sodium and rabbit polyclonal antibody against cyclooxygenase-2 (ab15191; Abcam, UK) were purchased from Beijing Diweile Biotechnology Co., Ltd. The SFZYD contained 11 herbs: *Foeniculi Fructus, Zingiberis Rhizoma, Corydalis Rhizoma, Myrrha, Rhizoma, Angelica Sinensis Radix, Radix Paeoniae Rubra, Cortex Cinnamomi, Typhae Pollen,* and *Trogopteri Feces*. The origins, medicinal parts, and weight ratios of these herbs have been described in a previous article [18]. We created the formulation as follows. In brief, all herbs except *Cortex Cinnamomi* were soaked in double-distilled water for 1 h and then decocted for 1 h. *Cortex Cinnamomi* was added last followed by decocting for 20 min. The final drug concentration was 1 g/mL after being concentrated by heating.

### 2.2. Animal Experiments and Groups

The animal procedures and care in this study were approved by the Animal Ethics Committee of North China University of Science and Technology (approval number 2016016). Six-to-eight-week-old female Sprague Dawley (SD) rats were purchased from the Laboratory Animal Center of the Academy of Military Medical Sciences. All rats were housed in a room (22°C ± 2°C) with a 12-hour light/dark cycle (7:00 AM to 7:00 PM), with ad libitum access to water and food (fundamental diet, Beijing Vital River Laboratory Animal Technology Co., Ltd) in groups of 4 animals per cage. Vaginal smears were taken daily from all rats after conventional adaptive feeding for 1 week. A total of 38 rats with normal estrous cycles were selected. Eight rats were randomly selected as the normal group, and the remainder were induced to develop endometriosis by autologously transplanting uterine tissue fragments onto the peritoneal wall. In brief, the remaining 30 rats were administered 0.2 mg of estradiol valerate 24 h before the surgery. Then, a 1.5–2 cm ventral midline incision was made after anesthetization induced by 2% pentobarbital sodium. The right side of the uterus was ligated and cut into 5 × 5 mm squares after rinsing in sterile phosphate-buffered saline (PBS). Each tissue square was sutured to the left peritoneal wall, where blood vessels are abundant, using a single nonabsorbable 5-0 polypropylene suture. After 4 weeks, rats in diestrus were selected for a second surgery to assess the implants. The transplanted endometrium was manifest as a sac-like shape or transparent nodule with fluid accumulation, which was regarded as successful induction [19]. The lesion sizes were measured using digital millimeter calipers.

Twenty-four rats with endometriotic lesions were randomized into three groups. The blank and model groups were treated with normal saline, the letrozole group with letrozole, and the SFZY group with SFZYD. Letrozole was given every day at a dose of 0.2 mg/kg/d. In the clinical practice of Chinese herbal medicine, the prescription dose of SFZY is usually 46.5 g of herbal materials per day. The dose of SFZYD given to rats was determined according to a formula that translates doses between different species based on the animal's body surface area. The administered dose of rats (g/kg) = $\left(46.5\times 0.9\times \sqrt[{3}]{{\left(x/60\right)}^{2}}\right)/x$ (*x*, the weight of rats; 60, the weight of adult females). Generally, the rats that weighed 300 g were given 1.2 g of SFZYD daily. Normal saline was administered at a dose of 1 mL/d in the blank and model groups. After 4 weeks of treatment, rats in diestrus were selected for their third surgery under anesthesia. Before anesthetization, fecal pellets were collected and immediately frozen in liquid nitrogen and then transferred to −80°C. The implants were measured with a digital millimeter caliper. Implant volume was calculated with the following formula: *V* (mm^3^) = 0.52 × length × width × height [19]. The implants were excised and fixed in 4% paraformaldehyde for immunohistochemistry.

### 2.3. Immunohistochemical Staining

After dewaxing, we placed the sections in 0.01 M citrate solution to retrieve the antigen at high temperature and pressure for 30 s followed by cooling to room temperature. Then, the sections were incubated with 3% hydrogen peroxide solution at room temperature for 20 min to interrupt peroxidase activity; we incubated them with goat serum at room temperature for 30 min for nonspecific blocking. Subsequently, we added primary antibody anti-cyclooxygenase-2 (1 : 200) to the sections and incubated them at 4°C overnight. Then, the secondary antibody (PV-9000, Zhongshan BioTech Co., Ltd., Beijing, China) was added, and sections were incubated at 37°C for 30 min, prior to diaminobenzidine (DAB) staining. We used an Olympus BX53 microscope (Japan) to obtain images, and Image-Pro Plus6 (Media Cybernetics, Inc., Rockville, MD, USA) was used to analyze the mean optical density (MOD) of each image.

### 2.4. Fecal DNA Extraction and Illumina MiSeq Sequencing

Genomic DNA was extracted from every fecal sample, and the purity and concentration of DNA were determined by agarose gel electrophoresis. Then, the sample was diluted to the concentration of 1 ng/*μ*l with sterile water, and the V3 and V4 regions of the 16s rDNA sequences were amplified using specific primers: forward primer-341F (5′-CCTAYGGGRBGCASCAG-3′) and reverse primer-806R (5′-GGACTACNNGGGTATCTAAT-3′). PCR reactions were performed using the following thermal profile: 1 min at 98°C for initial denaturation, 30 cycles of 10 s at 98°C for denaturation, 30 s at 50°C for annealing, and 30 s at 72°C for elongation followed by a final elongation step for 5 min at 72°C. We electrophoresed the samples to detect the PCR products using agarose gel as follows. The samples were mixed according to the concentration of PCR products, electrophoresis was performed by 1 ×  TAE in 2% agarose gel to purify the PCR products, and the target bands were recycled. A library was constructed following the manufacturer's instructions for the Ion Plus Fragment Library Kit and sequenced with an Ion S5TMXL sequencer.

### 2.5. Bioinformatics Analysis

Cutadapt (V1.9.1, http://cutadapt.readthedocs.io/en/stable/) was used for quality filtering to retrieve the raw reads [20]. We analyzed the raw reads using VSEARCH (https://github.com/torognes/vsearch/) to remove chimeric sequences and acquired the clean reads [21]. Sequences were clustered into operational taxonomic units (OTUs) at a similarity of 97% by Uparse software (Uparse v7.0.1001, http://www.drive5.com/uparse/) [22]. We conducted matching of OTUs to bacteria (the threshold value was 0.8–1 [23]) using the Mothur algorithm and SILVA132 reference database. Taxonomic information was obtained, and the species composition was classified at different levels, such as kingdom, phylum, class, order, family, genus, and species. We performed fast multisequence alignment using MUSCLE software (version 3.8.31, http://www.drive5.com/muscle/) to obtain the phylogenetic relationships of all OTU sequences [24]). A species accumulation boxplot was generated with R software (version 2.15.3), and *α*-diversity and *ß*-diversity were generated by QIIME (version 1.9.1). ACE, Chao1, observed-species, Shannon, Simpson, and PD-whole tree indices were then used to assess the richness and evenness of gut microbiota followed by a post hoc two-sided Wilcoxon–Mann–Whitney test for determining the statistical dependence among groups. The unweighted and weighted UniFrac approach was used to measure the *ß*-diversity among these four groups, and principal coordinate analysis (PCoA) was performed with R software (version 2.15.3) based on unweighted and weighted UniFrac data. AMOVA function was calculated using Mothur based on unweighted and weighted UniFrac algorithms to detect whether the species composition differences among groups were significant. We used the linear discriminant analysis (LDA) effect size (LEfSe) method to analyze the differences among groups at the phylum, class, order, family, and genus levels.

### 2.6. Statistical Analysis

Body weight gain and size and protein expression of endometriotic lesions were statistically analyzed using SPSS 23.0. Values are presented as means ± standard deviation (SD). All data were first assessed for a normal distribution and for homogeneity of variance. Size and protein expression of endometriotic lesions were compared using a paired-sample *t*-test or one-way analysis of variance (ANOVA) with Tukey's post hoc tests. Body weight gain repeated over time was compared using two-way ANOVA with post hoc Student's *t*-tests. *P* < 0.05 was considered statistically significant.

## 3. Results

### 3.1. Effects of Letrozole and SFZY on Endometriotic Lesion Growth and Weight Gain in Rats

**Figure 1:**
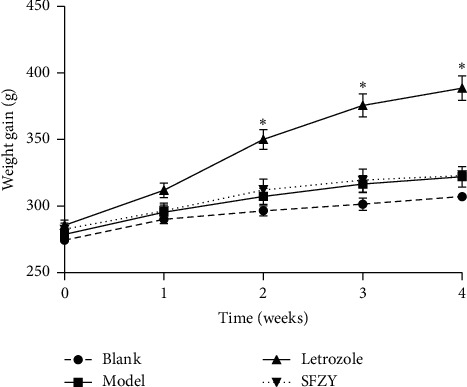
Body weight gain of rats in each group (two-way ANOVA with post hoc Student's *t*-tests, ^*∗*^*P* < 0.05).

As shown in Table 1, after treatment, the mean volume of endometriotic lesions decreased significantly in the letrozole group from 28.37 ± 18.49 to 3.57 ± 4.49 mm^3^ (*P* < 0.01) and in the SFZY group from 27.33 ± 15.24 to 8.05 ± 7.82 mm^3^ (*P* < 0.01); however, the mean volume of endometriotic implants in the model group increased significantly from 29.90 ± 13.34 to 45.26 ± 20.74 mm^3^ (*P* < 0.05). There was no difference between the letrozole and SFZY groups in lesion volume after medication administration (*P* > 0.05). As shown in Figure 1, the body weight gain in the letrozole group increased significantly compared with that in other groups (*P* < 0.05) from the second to the fourth weeks during treatment; there was no difference among the blank, model, or SFZY groups (*P* > 0.05).

### 3.2. Immunohistochemical Expression of Cyclooxygenase-2 in Endometriotic Lesions and Endometrium

**Figure 2:**
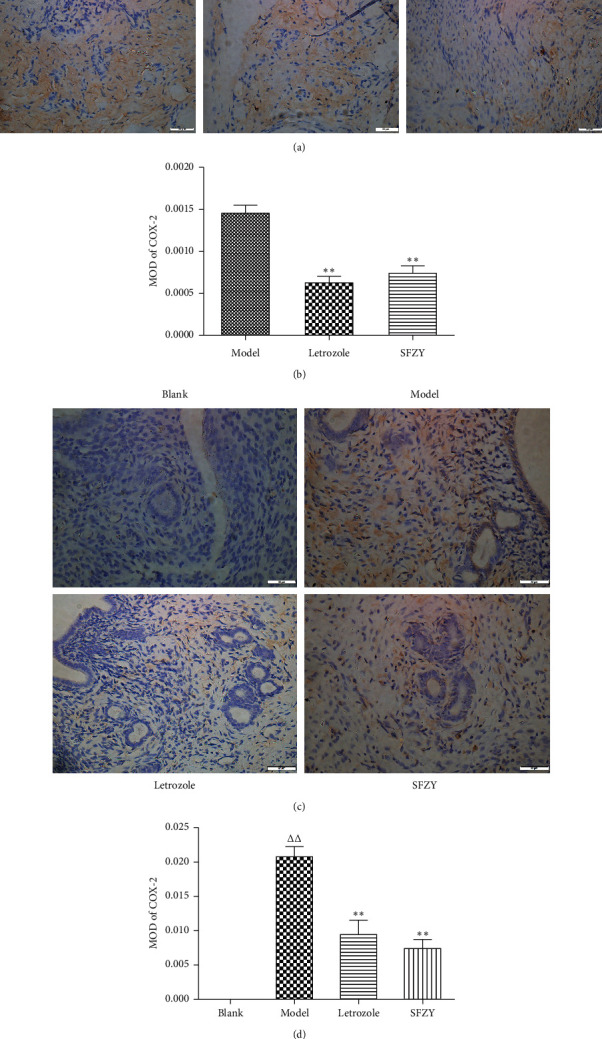
Representative images of immunohistochemical staining of COX-2 in endometriotic lesions and normal endometrium. (a) Sections stained for COX-2 in endometriotic lesions (magnification, ×400). (b) Mean optical density (MOD) values of the COX-2 expression (*n* = 6); one-way ANOVA with Tukey's test, ^*∗∗*^*P* < 0.01 compared with the model group. (c) Sections stained for COX-2 in the endometrium (magnification, ×400). (d) Mean optical density (MOD) values of the COX-2 expression (*n* = 6); one-way ANOVA with Tukey's test, ^ΔΔ^*P* < 0.01 compared with the blank group and ^*∗∗*^*P* < 0.01 compared with the model group.

According to immunohistochemical staining results (Figure 2), cyclooxygenase-2 (COX-2) protein expression in endometriotic lesions of the letrozole and SFZY groups was significantly lower than that of the model group (*P* < 0.01). COX-2 protein expression in the endometrium of the model group was significantly higher than that of the blank group (*P* < 0.01), and expression in endometriotic lesions of the letrozole and SFZY groups was significantly lower than that of the model group (*P* < 0.01).

### 3.3. Letrozole and SFZY Modulate the Composition of Gut Microbiota in Endometriotic Rats

**Figure 3:**
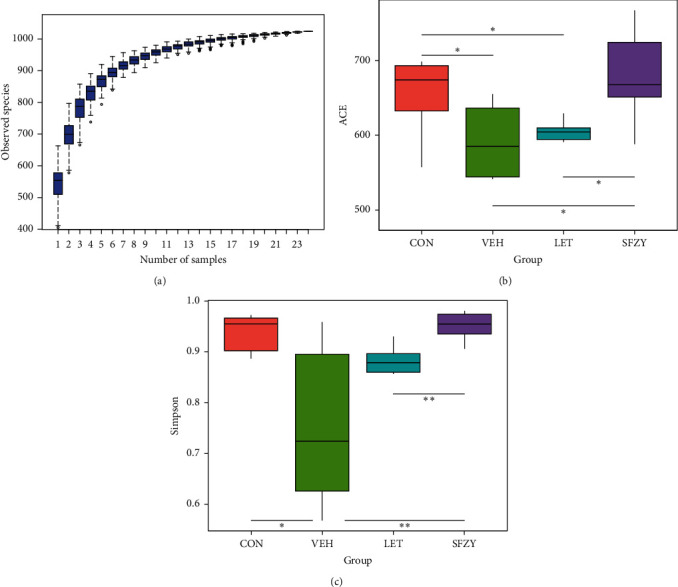
Gut microbiota composition is analyzed by *α*-diversity (*n* = 6). (a) A species accumulation boxplot is used to evaluate species richness and whether the sample size is sufficient. (b, c) ACE and Simpson analyses are used to evaluate community richness and diversity within each group (Kruskal–Wallis test, a nonparametric test is used; ^*∗*^*P* < 0.05 and ^*∗∗*^*P* < 0.01).

To detect the effects of letrozole and SFZY on gut microbial composition, we sequenced the 16S rRNA gene from DNA isolated from fecal samples from the four groups. An average of 85,049 reads were measured per sample, and 79,452 valid reads were obtained on average according to our quality blank measures. The effective rate of the quality blank was 93.49%, and sequences were clustered into OTUs within 97% sequence identity. We obtained a total of 1070 OTUs from 24 samples (Figure 3(a)).

We used the *α*-diversity to evaluate the richness and evenness of gut microbiota and ACE, Chao1, and observed-species indices to evaluate the richness of the gut microbiota. As shown in Figure 3(b), the ACE index in the model group was lower than that in the blank group (*P* < 0.05), which indicated that the richness in the model group was significantly lower than that in the blank. We observed no statistical difference for the Chao1 and observed-species indices between the blank and model groups. ACE, Chao1, and observed-species indices in the SFZY group that were similar to the blank group (*P* > 0.05) were significantly higher than those in the model and letrozole groups (*P* < 0.05). When we used Shannon, Simpson, and PD-whole tree indices to evaluate the richness and evenness of gut microbiota, the Simpson index in the model group was lower than that in the blank group (Figure 3(c), *P* < 0.05), which indicated that the richness and evenness in the model group were lower. No statistical difference was observed for the Shannon and PD-whole tree indices between the blank and model groups; the Shannon, Simpson, and PD-whole tree indices in the SFZY group were also similar to the blank (*P* > 0.05), but significantly higher than those in the model group (*P* < 0.01). These results demonstrated that the species richness and evenness of the model group were significantly lower than those of the blank group and like those of the letrozole group. The effects of STZY might therefore have improved species richness and evenness that were impaired by endometriosis.

**Figure 4:**
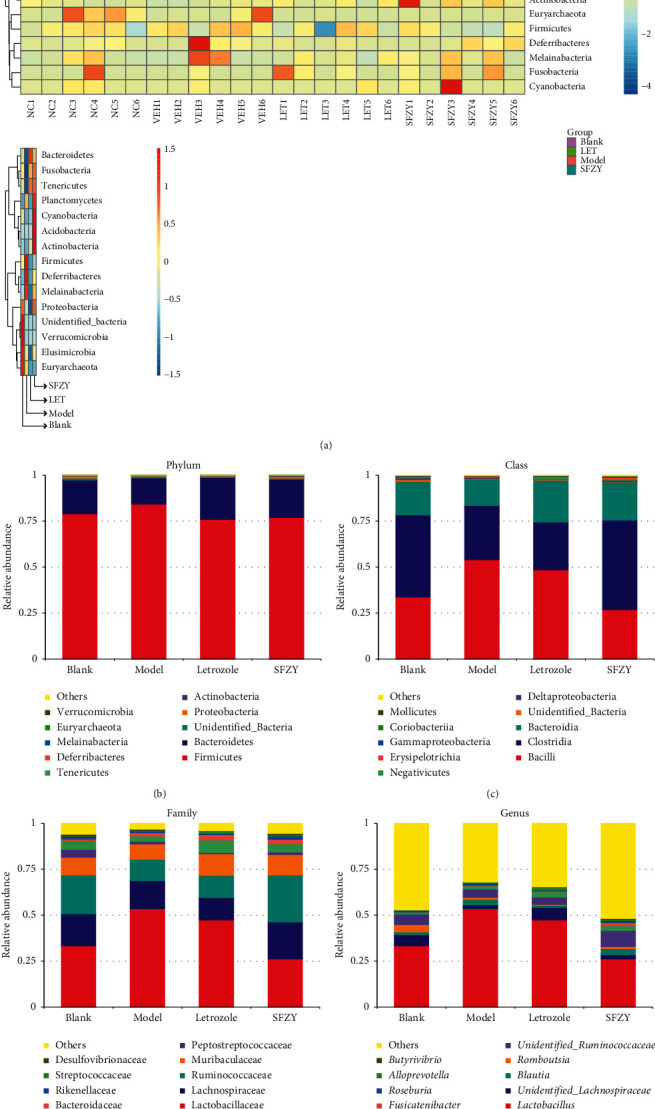
Analysis of species composition of gut microbiota among groups using OUT analysis (*n* = 6). (a) Heatmap representation of relative abundances of the phyla in fecal samples from each group. Microbiota distribution at the phylum level (b), class level (c), family level (d), and genus level (e).

To detect the relationship between microbiota composition and specific taxa, we profiled the phyla across samples in each group. As shown in Figure 4(a), fecal samples from model rats contained a higher abundance of *Firmicutes* and lower abundance of *Bacteroidetes* and *Proteobacteria* than samples from blank rats. Fecal samples from letrozole and SFZY groups contained a lower abundance of *Firmicutes* and higher abundance of *Bacteroidetes* than the model group. Fecal samples from letrozole rats contained a lower abundance of Proteobacteria than samples from the other three groups. We confirmed these findings by analyzing the 10 most-abundant OTUs at the phylum level (Figure 4(b)). At the class level, fecal samples from model rats contained a higher abundance of Bacilli and lower abundance of *Clostridia* and *Bacteroidia* than the blank rats. In contrast, fecal samples from letrozole rats contained lower abundances of Bacilli and *Clostridia* and a higher abundance of *Bacteroidia* than model rats. Fecal samples from SFZY rats also contained a lower abundance of Bacilli and higher abundance of *Bacteroidia* and *Clostridia* than samples from model rats (Figure 4(c)). At the family level, fecal samples from model and letrozole rats contained a higher abundance of *Lactobacillaceae* and lower abundances of *Ruminococcaceae* and *Peptostreptococcaceae* than from blank and SFZY rats (Figure 4(d)). At the genus level, fecal samples from model and letrozole rats contained a higher abundance of *Lactobacillus* than from either blank or SFZY rats (Figure 4(e)).

**Figure 5:**
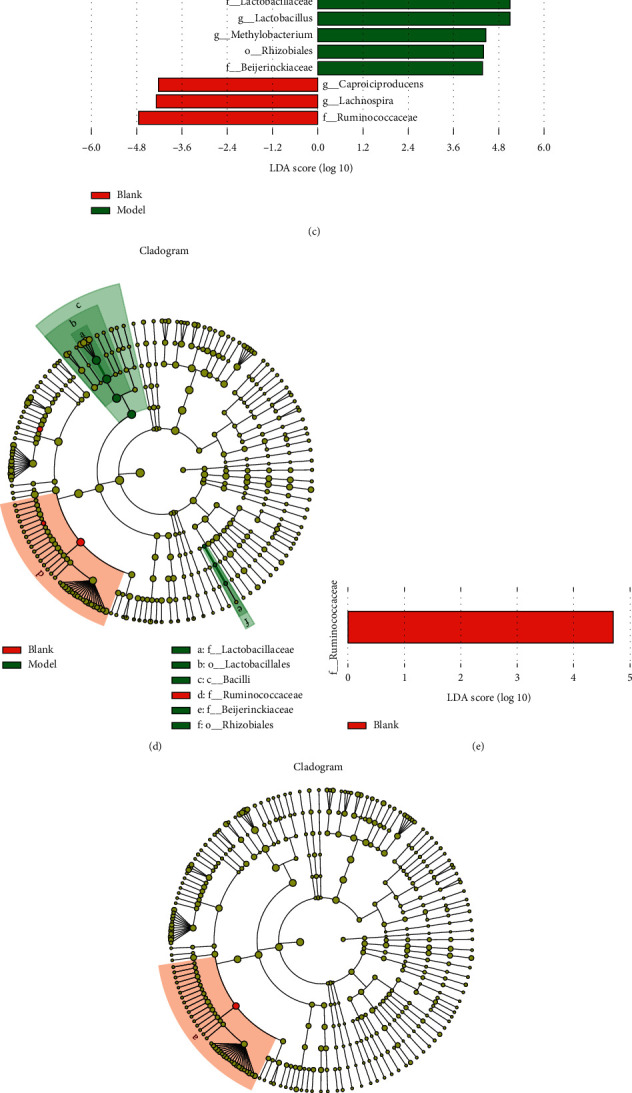
Comparison of gut microbiota among groups using PCoA and LEfSe analyses. (a, b) PCoA analysis based on unweighted and weighted UniFrac algorithms among groups. (c, d) Histogram of LDA scores and cladogram for differentially abundant genera between blank and model groups. (e, f) Histogram of LDA scores and cladogram for differentially abundant genera among blank, model, and cc vcx letrozole groups. (g, h) Histogram of LDA scores and cladogram for differentially abundant genera among blank, model, and SFZY groups.

To confirm the species composition differences among different groups, we profiled the *ß*-diversity based on a species-abundance profiling table. Principal coordinate analysis (PCoA) was performed based on unweighted and weighted UniFrac algorithms (Figures 5(a) and 5(b)), and we calculated the AMOVA function using Mothur based on these unweighted and weighted UniFrac algorithms to detect whether the species composition differences among groups were significant. According to AMOVA function analysis, the species composition from each group was significantly different (*P* < 0.01). To further identify specific species in each group, we performed LDA effect size (LEfSe). As shown in Figures 5(c) and 5(d), the histogram of LDA scores and cladogram indicated that the relative abundances of *Lactobacillaceae* and *Beijerinckiaceae* were enriched in model rats, and the relative abundance of *Ruminococcaceae* was higher in blank rats than in model rats at the family level. As shown in Figures 5(e) and 5(f), the histogram of LDA scores and cladogram indicated that the relative abundance of *Ruminococcaceae* was higher in blank rats than in model and letrozole rats at the family level. As shown in Figures 5(g) and 5(h), the histogram of LDA scores and cladogram indicated that the relative abundance of *Ruminococcaceae* was higher in SFZY rats than in model and blank rats at the family level, and the relative abundance of *Lactobacillus* was higher in model rats than in SFZY and blank rats at the genus level.

## 4. Discussion

There are nearly 10^14^ microbes in the human body, approximately 10 times the number of human cells, and the impact of the gut microbiota on human health and disease has attracted increasing scientific interest [25–28] in recent years, including in areas such as obesity, inflammatory diseases, cardiovascular diseases, and tumors. The gut microbiota is believed to be involved in the pathologic process of EMs [29]. Existing experimental studies primarily focus on mouse models of endometriosis. For example, Yuan et al. demonstrated that the *Firmicutes/Bacteroidetes* ratio was elevated in mice with endometriosis, as were the abundances of *Firmicutes* and *Bacteroidetes* in the gut microbiota flora of mice 42 days after modeled induction [15]; this is consistent with our study. In contrast, Josefine et al. investigated the gut microbiota of mice 21 days after inducing endometriosis and found that there was no effect on the microbiota [30]. Our contrasting conclusions may be due to the difference in sampling times. Most investigators posit that, under conditions of high fat, the gut microbiota of rodents increases the abundance of *Firmicutes* and decreases the abundance of *Bacteroidetes*. The increased *Firmicutes/Bacteroidetes* ratio in the gut appears to weaken the barrier function of the intestinal epithelium and increase LPS levels in plasma, which is conducive to the formation of endotoxemia [31]. The translocation of LPS from the intestinal epithelium to the pelvic cavity is then conducive to the maintenance of pelvic inflammation [13], which might provide a favorable environment for ectopic endometrial implantation. In addition, Sangappa et al.'s study suggested that the abundance of *Firmicutes* in the gut of mice with endometriosis decreased while that of *Bacteroidetes* increased [16], which contradicts the conclusions in our current study. As such, the specific role of gut microbiota in the development of endometriosis needs to be studied and elucidated further. We also found a decrease in the abundance of *Ruminococcaceae* bacteria in the model group. Interestingly, some studies have suggested that *Ruminococcaceae* is negatively correlated with apoptosis of intestinal epithelial cells and IL-6 levels in mice [32], indicating that decreased abundance of *Ruminococcaceae* may aggravate pelvic inflammation.

Our research results showed that both letrozole and SFZYD inhibited the progression of ectopic lesions in rats with endometriosis and reduced inflammatory response by inhibiting the expression of COX-2 in ectopic and eutopic endometrial tissues. After comparing the differences in gut microbiota of the four groups of rats, we found that both medications reduce the *Firmicutes/Bacteroidetes* ratio, which might be one of the mechanisms underlying the reduction in the inflammatory response in rats with endometriosis. Based on previous studies, an increase in the *Firmicutes/Bacteroidetes* ratio is likely related to a low-grade inflammation that not only affects the gut but also affects other organs [33]. We therefore speculated that the reduction in the *Firmicutes/Bacteroidetes* ratio by these two drugs may be beneficial in reducing inflammation induced by endometriosis. However, in the present study, we could not prove that the correlation between gut microbiota and inflammation was directly caused by endometriosis; this needs to be confirmed further by fecal-transplant experiments.

After treatment, estrous cyclicity in the letrozole group ceased, with the rats remaining in diestrus. To ensure the accuracy of our experimental conclusions, we sampled the other groups of rats on diestrus. In animal studies, long-term administration of letrozole has been found to lead to obesity and polycystic ovarian changes such that letrozole has been used to induce polycystic ovary syndrome in animal models [34, 35]. In clinical trials, the major adverse reactions to letrozole in the treatment of endometriosis were related to the low concentrations of circulating estrogen, i.e., vaginal dryness, hot flashes, and osteoporosis; these reactions have limited letrozole's clinical application [25]. The effect of letrozole on gut microbiota is mostly found in studies involving animal models of polycystic ovarian syndrome (PCOS). Scott et al. showed that, in a letrozole-induced mouse model, the abundance of *Firmicutes* in the gut increased while that of *Bacteroidetes* decreased [36], which is inconsistent with the conclusions of the present study. The dosage of letrozole in Scott et al.'s study was, however, much higher than in our study, and this may be a reason for the difference in the conclusions drawn. Our analysis of the *α*-diversity confirmed that letrozole impaired the richness and evenness of gut microbiota in rats and diminished the abundance of *Ruminococcaceae*. In addition, when we evaluated the changes in body weights of rats in each group, we demonstrated that the upregulation of the *Firmicutes*/*Bacteroidetes* ratio was generally associated with obesity [25]. Although rat body weight in the letrozole group increased significantly, the *Firmicutes/Bacteroidetes* ratio in this group was not significantly different from the blank group. Our LEfSe comparison of specific taxa between the letrozole and blank groups showed that the increased abundance of *Streptococcaceae* in the gut of letrozole-treated rats was also related to rat obesity [37]. Therefore, the effect of letrozole on the gut microbiota of clinical patients needs to be further evaluated.

In the SFZY group, the abundance of *Bacilli* and *Ruminococcaceae* increased markedly at the class level. Based on the *ß*-diversity indices, the comparison between the blank group and the SFZY group confirmed that there was no statistically significant difference between the two groups, suggesting that SFZYD normalized the gut microbiota in rats with endometriosis. This included reducing the *Firmicutes/Bacteroidetes* ratio and increasing the abundance of *Ruminococcaceae*, which could restore the impaired intestinal barrier function and indirectly alleviate the inflammatory response of rats with endometriosis as we have discussed above. It is worth noting that SFZYD has been shown to reduce inflammation and promote lipid metabolism in a high-fat diet mouse model [38]. In addition, the network pharmacology analysis showed that the lipid metabolism-related pathway constituted potential protein targets of SFZYD [39]. The abundance of *Ruminococcaceae* and *Prevotellaceae* was increased significantly in SFZY rats relative to model rats, and these probiotics decompose complex carbohydrates into short-chain fatty acids (SCFAs) that are energy sources for intestinal epithelial cells and protective factors in gut inflammation [40, 41]. As a consequence, SFZY would likely restore impaired gut microbiota and then promote SCFA production to improve the gut barrier, which would play a beneficial role in endometriosis treatment by reducing inflammation in the ectopic endometrium and in the pelvic cavity.

## 5. Conclusions

In this study, we confirmed that modulation of gut microbiota exists in endometriotic rats and that it is principally characterized by an augmented *Firmicutes/Bacteroidetes* ratio and reduced abundance of *Ruminococcaceae*. We postulate that both letrozole and SFZYD reduced the inflammatory response of ectopic and eutopic endometrial tissues, which might be related to the decrease in the *Firmicutes/Bacteroidetes* ratio.

## Figures and Tables

**Table 1:** Comparison of mean endometriotic implant volumes in the groups (*n* = 8).

Group	Before medication (mm^3^)	After medication (mm^3^)
Model	29.90 ± 13.34	45.26 ± 20.74^*∗*^
Letrozole	28.37 ± 18.49	3.57 ± 4.49Δ^*∗∗*^
SFZY	27.33 ± 15.24	8.05 ± 7.82Δ^*∗∗*^

*Note.* ^Δ^ *P* < 0.01 compared with the model group after medication, ^*∗*^*P* < 0.05 compared with the same group before medication, and ^*∗∗*^*P* < 0.01 compared with the same group before medication.

## Data Availability

The data used to support the findings of this study are available upon request from the corresponding author and the first author.
